# Serum Metabolite Biomarkers Discriminate Healthy Smokers from COPD Smokers

**DOI:** 10.1371/journal.pone.0143937

**Published:** 2015-12-16

**Authors:** Qiuying Chen, Ruba S. Deeb, Yuliang Ma, Michelle R. Staudt, Ronald G. Crystal, Steven S. Gross

**Affiliations:** 1 Department of Pharmacology, Weill Cornell Medicine, 1300 York Avenue, New York, NY, 10065, United States of America; 2 Department of Genetic Medicine, Weill Cornell Medicine, 1300 York Avenue, New York, NY, 10065, United States of America; Georgia Regents University, UNITED STATES

## Abstract

COPD (chronic obstructive pulmonary disease) is defined by a fixed expiratory airflow obstruction associated with disordered airways and alveolar destruction. COPD is caused by cigarette smoking and is the third greatest cause of mortality in the US. Forced expiratory volume in 1 second (FEV1) is the only validated clinical marker of COPD, but it correlates poorly with clinical features and is not sensitive enough to predict the early onset of disease. Using LC/MS global untargeted metabolite profiling of serum samples from a well-defined cohort of healthy smokers (n = 37), COPD smokers (n = 41) and non-smokers (n = 37), we sought to discover serum metabolic markers with known and/or unknown molecular identities that are associated with early-onset COPD. A total of 1,181 distinct molecular ions were detected in 95% of sera from all study subjects and 23 were found to be differentially-expressed in COPD-smokers vs. healthy-smokers. These 23 putative biomarkers were differentially-correlated with lung function parameters and used to generate a COPD prediction model possessing 87.8% sensitivity and 86.5% specificity. In an independent validation set, this model correctly predicted COPD in 8/10 individuals. These serum biomarkers included myoinositol, glycerophopshoinositol, fumarate, cysteinesulfonic acid, a modified version of fibrinogen peptide B (mFBP), and three doubly-charged peptides with undefined sequence that significantly and positively correlate with mFBP levels. Together, elevated levels of serum mFBP and additional disease-associated biomarkers point to a role for chronic inflammation, thrombosis, and oxidative stress in remodeling of the COPD airways. Serum metabolite biomarkers offer a promising and accessible window for recognition of early-stage COPD.

## Introduction

Chronic obstructive pulmonary disease (COPD) is a debilitating cigarette smoking-associated disease and the third most common cause of death in the US. The natural history of COPD is marked by episodes of exacerbation, morbidity and ultimately, mortality [1]. Other than O_2_ inhalation, there are no therapies that diminish mortality of COPD [2]. To promote COPD education and set universal standards of treatment, the Global Initiative for Chronic Obstructive Lung Disease (GOLD) was initiated in 1998, and updated every year, incorporating the latest science for improving the lives of COPD patients around the world [3].

COPD is characterized by progressive restriction to expiratory airflow [4]. The forced expiratory volume in 1 second (FEV1) both defines and characterizes the severity of the disease, and is a well-known predictor of mortality [5]. However, COPD manifests itself with multiple phenotypes that cannot be identified through measurement of lung function alone. The importance of COPD risk assessment, phenotype identification, and early prognosis of exacerbation periods magnify the need for validated biomarkers in COPD [6]. A variety of promising biomarkers have been reported for COPD [7], including blood proteins [8–39], miRNAs [40, 41], and small molecule metabolites [42–50]. These proposed biomarkers are specifically associated with different aspects of COPD pathophysiology. Indeed, inflammatory markers in blood such as increased C-reactive protein [21, 33], fibrinogen [8, 9], IL-6 [34–36], IL-8 [37] and tumor necrosis factor alpha [38] have been linked to exacerbations and mortality, while reduced CC-16 [22] and elevated adiponectin [30, 31, 39] have been linked to lung function deficits and severity of emphysema. Notwithstanding rapidly expanding efforts to identify COPD biomarkers, especially blood proteins, no single marker has been adopted for routine clinical use to date.

Radiological markers, such as quantitative CT imaging have been used to define the COPD-related phenotype in smokers [51]. Gene expression [52, 53] and analysis of molecules in exhaled breath [54–56] have also been applied for potential discovery of COPD biomarkers. Most recently, metabolomics-based metabolite profiling has begun to attract attention as a biomarker discovery approach [57, 58]. Indeed, metabolites are the ultimate readout of disease phenotype and accordingly, metabolomics has proven to be a powerful tool for probing complex biological mixtures to discover changes in metabolite levels that arise from drug treatments, gene mutations and disease states. Paige et.al [48] analyzed plasma metabolite profiles from healthy non-smokers, smokers with emphysema, and smokers without emphysema, using ultra high performance liquid chromatography/quadrupole–time-of-flight mass spectrometry (UPLC–QTOF). Based on observed differences in metabolite profiles, 12 unknown target metabolites were used to generate a predictive model that identified emphysema patients with 90% sensitivity [48]. A recent gas chromatography-mass spectrometry (GC-MS) based plasma metabolite profiling was applied to effectively predict the presence of cancer in 10 out of 10 lung cancer patients, 8 out of 8 non-smokers, 8 out of 9 COPD smokers and 5 out of 10 healthy smokers, based on 32 differentially-expressed metabolites across the 4 patient groups [47]. Of these 32 putative metabolite biomarkers, 21 were of unknown molecular identity, but were defined by their accurate mass and chromatographic retention times.

In attempt to uncover biomarkers for early-stage COPD, we performed untargeted LC/MS profiling of a cohort of 118 human subjects comprised of healthy smokers, COPD smokers and healthy non-smokers, all with well-characterized clinical lung function parameters. The COPD smokers used in this study were classified as mild, belonging to GOLD I /II disease severity groups [3]. We surveyed relative levels of 1,181 molecular ions that could be measured in 95% of patient serum samples to generate a COPD prediction model with 87.8% sensitivity and 86.5% specificity. Efficacy of the model for predicting COPD was found to be 80% in a follow-on study of a small independent cohort. Differentially-upregulated molecules in COPD smokers vs. healthy smokers included a modified form of fibrinogen peptide B along with 3 additional peptides. To our knowledge, this is the first discovery of significantly elevated (greater than 2-fold) serum peptides in early-stage COPD patients with predictive potential. These results point to an intriguing linkage between thrombosis, cell signaling aberrations and early onset of COPD.

## Materials and Methods

### Human patient cohort

Serum samples from an initial cohort of 118 human subjects were categorized into 3 groups: healthy nonsmokers (n = 37), healthy smokers (n = 40) and COPD smokers (n = 41). These samples were used in attempt to identify small molecule biomarkers (50–1000 Da) that significantly distinguish COPD smokers from healthy smokers and nonsmokers. The validity of inferred serum COPD biomarkers was further assessed in an independent study cohort comprising healthy nonsmokers (n = 10), healthy smokers (n = 9), COPD smokers (n = 10) and COPD subjects who had quit smoking for > 3 months (n = 19). Serum samples were obtained in the morning following overnight fasting. All subjects were phenotyped with regard to medical history, physical exam, routine blood and urine tests, urine nicotine/cotinine levels, lung function (forced vital capacity (FVC), forced expiratory volume in 1 sec (FEV1), FEV1/FVC, total lung capacity and diffusing capacity and chest high-resolution tomography (HRCT). Serum samples from 3 smokers (2 males and 1 female) were not acquired for LC/MS negative ion mode due to a sample handling error (See LC/MS methods for description). All COPD subjects were categorized as GOLD I and II in terms of severity, according to post-bronchodilator lung function defined by the GOLD classification [3].

### Reagents

LC-MS grade acetonitrile (ACN), isopropanol (IPA) and methanol (MeOH) were purchased from Fischer Scientific. High purity deionized water (ddH_2_O) was filtered from Millipore (18 OMG). OmniTrace glacial acetic acid and ammonium hydroxide were obtained from EMD Chemicals. Ammonium acetate and all other chemicals and standards were obtained from Sigma Aldrich in the best available grade.

### Serum metabolite extraction and metabolomic data acquisition

Serum metabolites were extracted by addition to 1 part serum to 20 parts (vol:vol) 70% acetonitrile in ddH_2_O containing 0.2% ammonium hydroxide. The mixture was briefly vortexed and then centrifuged for 5 min at 16,000 × g to pellet precipitated proteins. An aliquot of the resulting extract (3 μl) was subjected to untargeted metabolite profiling using LC/MS and both positive and negative ion monitoring. The sequence of analyzed samples was randomized to eliminate sample order bias of results. Additionally, to minimize bias that may be introduced by drifting of chromatographic retention times and detected mass measurements with large sample runs, a pooled extract from all serum samples was prepared, stored in single-thaw aliquots, and used as a quality control to correct for potential day-to-day and batch-to-batch LC/MS data drift. One quality control sample was analyzed every 6 study samples and flanking quality control results were used to normalize the group of intervening samples.

### LC/MS metabolomics platform for untargeted metabolite profiling

Serum extracts were analyzed by LC/MS as described previously [59, 60] using a platform comprised of an Agilent Model 1200 liquid chromatography system coupled to an Agilent 6230 time-of-flight MS analyzer. Chromatography of metabolites was performed using aqueous normal phase (ANP) gradient separation, on a *Diamond Hydride* column (Microsolv). Mobile phases consisted of: (A) 50% isopropanol, containing 0.025% acetic acid, and (B) 90% acetonitrile containing 5 mM ammonium acetate. To eliminate the interference of metal ions on the chromatographic peak integrity and electrospray ionization, EDTA was added to the mobile phase at a final concentration of 6 μM. The following gradient was applied: 0–1.0 min, 99%B; 1.0–15.0 min, to 20%B; 15.0 to 29.0, 0%B; 29.1 to 37min, 99%B. Raw data were analyzed using MassHunter Profinder 6.0 and MassProfiler Professional 13.0 software package (Agilent technologies). Unpaired t-tests (p<0.05) with Bonferroni correction for multiple group comparisons and ANOVA were used to determine significant differences between groups.

### Differentially expressed metabolite identification

To ascertain the identities of differentially expressed metabolites (P<0.05) among COPD smokers, healthy smokers and healthy non-smokers, LC/MS data were searched against an in-house annotated *METLIN* Personal Metabolite Database (Agilent Technologies), based on accurate monoisotopic neutral masses (<5 ppm) and chromatographic retention times. A molecular formula generator (MFG) algorithm in MPP was used to generate and score empirical molecular formulae based on a weighted consideration of monoisotopic mass accuracy, isotope abundance ratios, and spacing between isotope peaks. A tentative compound ID was assigned when *METLIN* and MFG scores concurred for a given candidate molecule. Tentatively assigned molecules were verified based on a match of LC retention times and/or MS/MS fragmentation spectra to that of pure molecule standards in our progressively growing in-house database.

## Results

### Clinical characteristics of human subjects

Clinical characteristics of the initial study group are summarized in **Table 1**. A total of 118 serum samples were collected from 92 males and 26 females. The nonsmoker group comprised a balanced number of male and female patients, whereas the healthy smoker and COPD smoker groups were dominated by male subjects. Baseline parameters were similar in BMI and ethnicity for all groups. Both healthy smokers and COPD smokers had a comparable cigarette consumption rate and smoking history. COPD subjects on average were older than healthy smokers and non-smokers.

**Table 1:** Human subjects and associated clinical parameters^1^.

	Non-smokers	Healthy smokers	COPD smokers
**Number of subjects**	37	40	41
**Gender**	19M, 18F	35M, 5F	38M, 3F
**Ethnicity**	14 His, 13 Afr, 10 Eur	14 His, 11 Afr, 5 Eur	9 His, 23 Afr, 8 Eur, 1 mixed
**Packs per year**	n/a	21.5 ± 11.3	30.6 ± 1.7
**Packs per day**	n/a	0.9 ± 0.6	0.8 ± 0.5
**Age of initiation**	n/a	15.2 ± 2.9	16.7 ± 3.3
**Age**	39.5 ± 13.0	41.8 ± 10.1	53.2 ± 7.7
**BMI**	26.6 ± 5.0	26.9 ± 4.9	25.6 ± 3.6
**FEV1%**	104.0 ± 11.5	105.5 ± 11.0	77.7 ± 18.9
**FVC%**	108.0 ± 11.3	110.5 ± 11.0	106.0 ± 16.4
**FEV1/FVC%**	80.0 ± 5.7	77.9 ± 4.3	58.5 ± 7.6
**TLC**	97.7 ± 15.7	93.8 ± 9.2	101.6 ±14.5
**DLCO**	90.4 ± 11.4	87.2 ± 8.5	68.5 ±14.3
**COHb**	1.6 ± 0.8	2.9 ± 1.2	3.3 ± 1.2
**Urine cotinine**	n/a	1687.0 ±1073.4	1619.5 ±885.1

^1^All values were presented as mean ± standard deviation

M = Male, F = Female; His: Hispanic ethnicity, Afr: African ethnicity; Eur: European ethnicity; FEV1: forced expiratory value in 1 second; FVC: functional vital capacity; TLC: total lung capacity; DLCO, diffusing capacity of the lung for carbon monoxide. COHb: carboxylhemoglobin. All lung function parameters are presented as % predicted except for FEV1/FVC which is expressed as % observed. N/A = not applicable.

Urine cotinine, the nicotine metabolite, and blood COHb were at comparable levels for healthy smokers and COPD smokers and, as expected, low or undetected in non-smokers. Compared to healthy non-smokers and healthy smokers, COPD smokers had significantly decreased DLCO and FEV1/FVC ratio, the basis for clinical diagnosis of COPD. DLCO and FEV1/FVC were significantly correlated with each other (Pearson correlation coefficient R = 0.62, P < 6.3 x e^-14^) and readily discriminated healthy smokers from COPD smokers. Total lung capacity showed no difference between COPD smokers and nonsmokers. All COPD smokers were classified as belonging to the least severe, GOLD I and GOLD II disease groups Given that healthy smokers and COPD smokers had an indistinguishable cigarette exposure and smoking history, comparison of plasma metabolites between these groups should allow for discovery of biomarkers with potential to distinguish the groups and predict health outcomes.

### Serum metabolite profile of non-smokers, healthy smokers and COPD smokers

COPD is routinely diagnosed based on clinical parameters related to lung airway function (e.g. FEV1/FVC%). However, such clinical parameters do not directly correlate with the overall health status of patients. Moreover, measurements of lung function provide little prognostic information regarding anticipated disease severity. In contrast, circulating serum metabolites offer a potential window into systemic physiology/pathophysiology and the possibility of informative and predictive COPD biomarkers. To compensate for the limited prognostic potential of clinical lung parameters, we sought to identify serum metabolites that may inform on COPD health status and outcomes.

**Fig 1:**
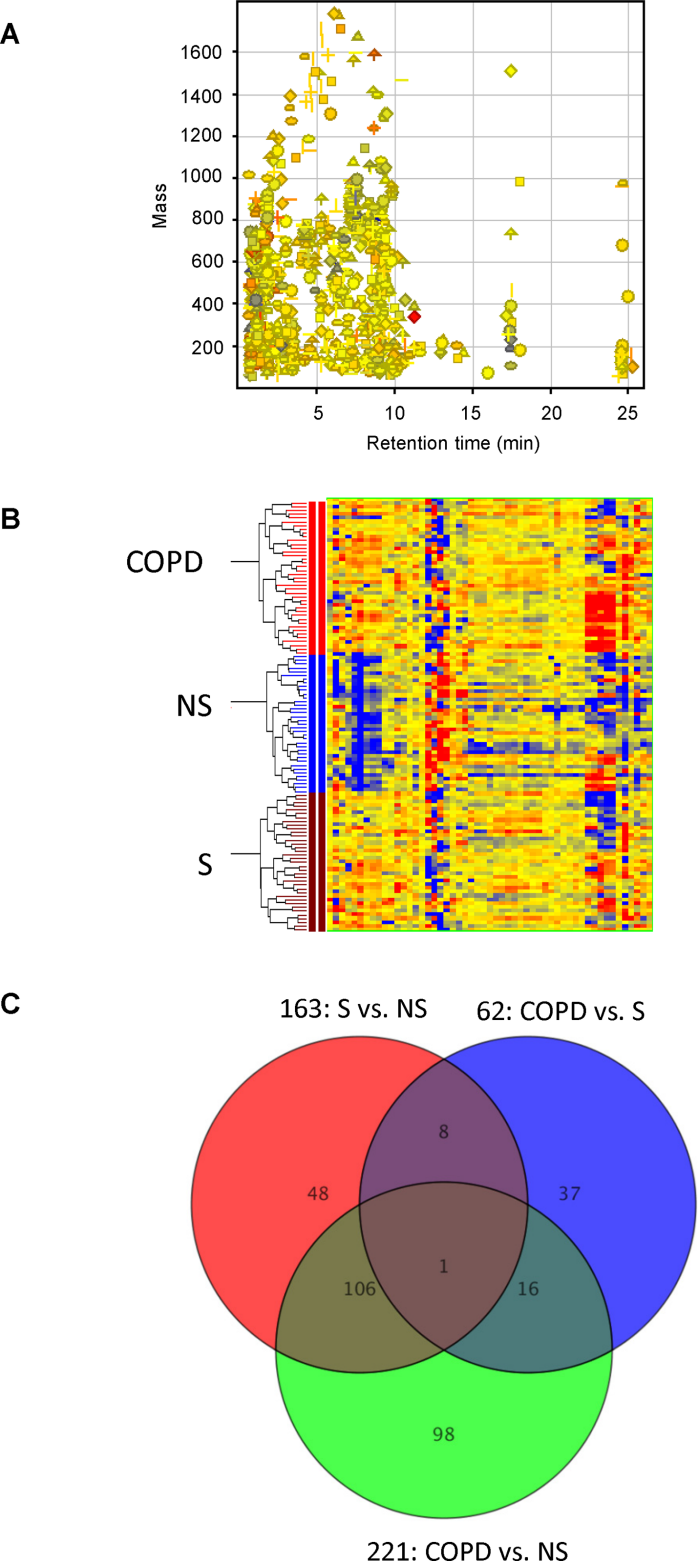
Untargeted metabolite profiling identifies differentially-expressed serum metabolites among COPD smokers, healthy smokers, and non-smokers (P<0.05). (A) A total of 1,181 aligned molecular features detected as positive and negative ions were quantified in all 115 serum samples by untargeted molecular feature extraction. (B) Heat map from hierarchical clustering analysis, showing 314 differentially-expressed metabolites among COPD smokers, healthy smokers and non-smokers. The relative level of expression of each metabolite is represented by a color coded heat map, where red represents an expression higher than the mean and blue represents an expression below the mean values. (C) Venn Diagram depicts shared metabolite changes among COPD smokers, healthy smokers and non-smokers (p < 0.05). Of these, 62 metabolites were significantly different (P<0.05) between healthy smokers and COPD smokers. And 17 of these 62 metabolites were differentially-expressed in COPD smokers compared to both healthy smokers and non-smokers, but showed no difference between healthy smokers and non-smokers.

Applying an untargeted LC/MS-based metabolomics platform that uses aqueous normal phase chromatographic separation coupled to time-of-flight MS, we successfully quantified the relative levels of 586 positive ions and 635 negative ions in 95% of all serum samples studied (including quality control samples). Combining positive and negative ions, after excluding duplicate features measured in both MS detection modes, 1,181 total aligned molecular features in the range of 50–1,000 Da were quantified in all serum samples **(Fig 1A)**. To account for day-to-day and batch-to-batch LC/MS instrument drift, metabolite stability and other experimental factors that can contribute to systematic error, we normalized individual serum metabolite measurements from all subjects based on levels quantified in flanking quality control samples. This normalization procedure enabled the comparative analysis of data from a relatively large number of clinical samples, quantified on different occasions.

Unsupervised hierarchical cluster analysis (HCA) revealed a clear and reproducible pattern of within-group metabolite expression similarities and between-group differences **(Fig 1B).** A Venn diagram, depicted in **Fig 1C**, contrasts the number of differentially expressed metabolites and shared metabolite changes among healthy non-smokers, healthy smokers and COPD smokers. Of 1,181 features quantified in 95% of samples, 62 metabolites were found to be significantly different in abundance (P<0.05) between healthy smokers and COPD smokers. Of these, 17 metabolites were differentially-expressed (up or down) in COPD smokers compared to both healthy smokers and healthy non-smokers, but also showed no difference between healthy smokers and healthy non-smokers (i.e., metabolites that distinguish COPD smokers from both healthy smokers and healthy non-smokers). Regardless of whether smokers were healthy or had COPD, smoking itself was associated with 107 serum metabolite changes, relative to non-smokers. Among these smoking-associated serum metabolites, 20 were unknowns that were elevated by 1.5-fold or greater in both healthy and COPD smokers, compared to non-smokers (P<0.05, **Table 2**). These 20 unknowns were defined by their accurate neutral mass and chromatographic retention time on ANP chromatographic matrix. As expected, the abundant nicotine metabolites, cotinine and hydroxy-cotinine, were dramatically increased in serum from both healthy and COPD smoker groups. Additionally, smoking was associated with altered levels of serum phospholipids and bile acids, but the extent of these changes were undistinguishable between healthy smokers and COPD smokers.

**Table 2:** Differential metabolites between COPD smoker and healthy smoker compared to non-smokers.

METABOLITE	Mass	RT	P	FC	P	FC	P	FC
			S vs NS	S vs NS	COPD vs NS	COPD vs NS	COPD vs S	COPD vs S
**Cotinine**	176.093	3.67	1.54E-14	23.27	5.84E-18	24.09	7.81E-01	1.04
**3-Hydroxycotinine**	192.092	3.45	1.99E-13	4.85	4.23E-13	5.51	2.97E-01	1.14
**Unknown 1**	742.23	0.71	1.36E-03	3.36	2.84E-02	2.5	3.54E-01	0.74
**Quinic acid**	192.061	4.41	6.98E-03	2.60	5.33E-04	3.77	1.72E-01	1.45
**lysoPI(20:0)**	628.36	4.47	3.85E-02	2.19	5.14E-02	2.23	9.62E-01	1.02
**PI(32:2)**	806.506	1.8	1.40E-02	2.11	8.52E-03	1.78	4.83E-01	0.84
**Glycochenodeoxycholic acid 3-glucuronide**	625.345	6.53	1.81E-03	2.00	7.43E-02	2.23	7.49E-01	1.11
**PI(32:1)**	808.511	1.84	6.57E-03	1.89	1.90E-02	1.47	2.27E-01	0.78
**5-Acetylamino-6-formylamino-3-methyluracil**	226.072	0.69	3.85E-03	1.89	2.13E-02	1.42	1.34E-01	0.75
**Unknown2**	600.451	1.02	1.39E-02	1.83	4.00E-03	1.88	9.00E-01	1.03
**PI(32:0)**	810.516	1.84	2.73E-03	1.81	1.33E-02	1.47	2.30E-01	0.81
**Glycochenodeoxycholate-3-sulfate**	529.27	1.61	1.63E-02	1.65	7.96E-02	1.7	9.09E-01	1.03
**PE(35:1)**	731.546	7.64	7.91E-03	1.65	7.27E-03	1.46	4.52E-01	0.88
**Unknown3**	200.014	0.72	3.04E-02	1.61	4.91E-03	2.52	7.60E-02	1.57
**1α,25-dihydroxy-23-thiavitamin D3**	434.296	1.77	1.53E-03	1.54	1.93E-03	1.52	9.34E-01	0.99
**PI(40:4)**	914.583	1.84	5.95E-04	1.54	2.38E-04	1.5	7.92E-01	0.97
**Unknown**	602.468	1.03	3.24E-02	1.52	1.75E-03	1.63	6.95E-01	1.07
**6-methyltetrahydropterin**	181.098	1.19	2.58E-05	1.51	2.85E-07	1.8	3.19E-02	1.19
**PE(33:1)**	703.516	7.71	2.78E-02	1.51	1.90E-02	1.43	7.24E-01	0.94
**PI(34:1)**	836.539	1.85	2.67E-03	1.51	1.68E-02	1.3	2.42E-01	0.86

^1^RT = retention time

P = p value; S = smokers, NS = nonsmoker, FC = fold-change

### COPD-specific serum metabolite changes

**Fig 2:**
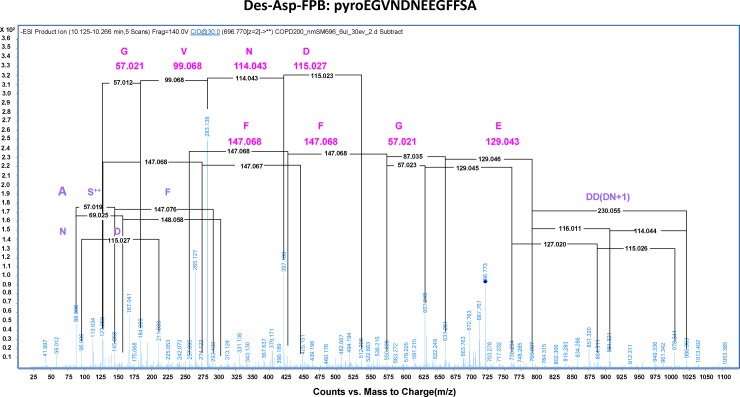
MS/MS fragmentation of a doubly-charged ion ([M+2H] = 697.97 Da) reveals the sequence of peptide as pyroEGVNDNEEGFFSA. Fragments derived from ion fragmentation at 30 EV match the amino acid sequence of fibrinogen peptide B with truncated C-terminal arginine and N-terminal amino acid modified as pyroglutamate.

**Fig 3:**
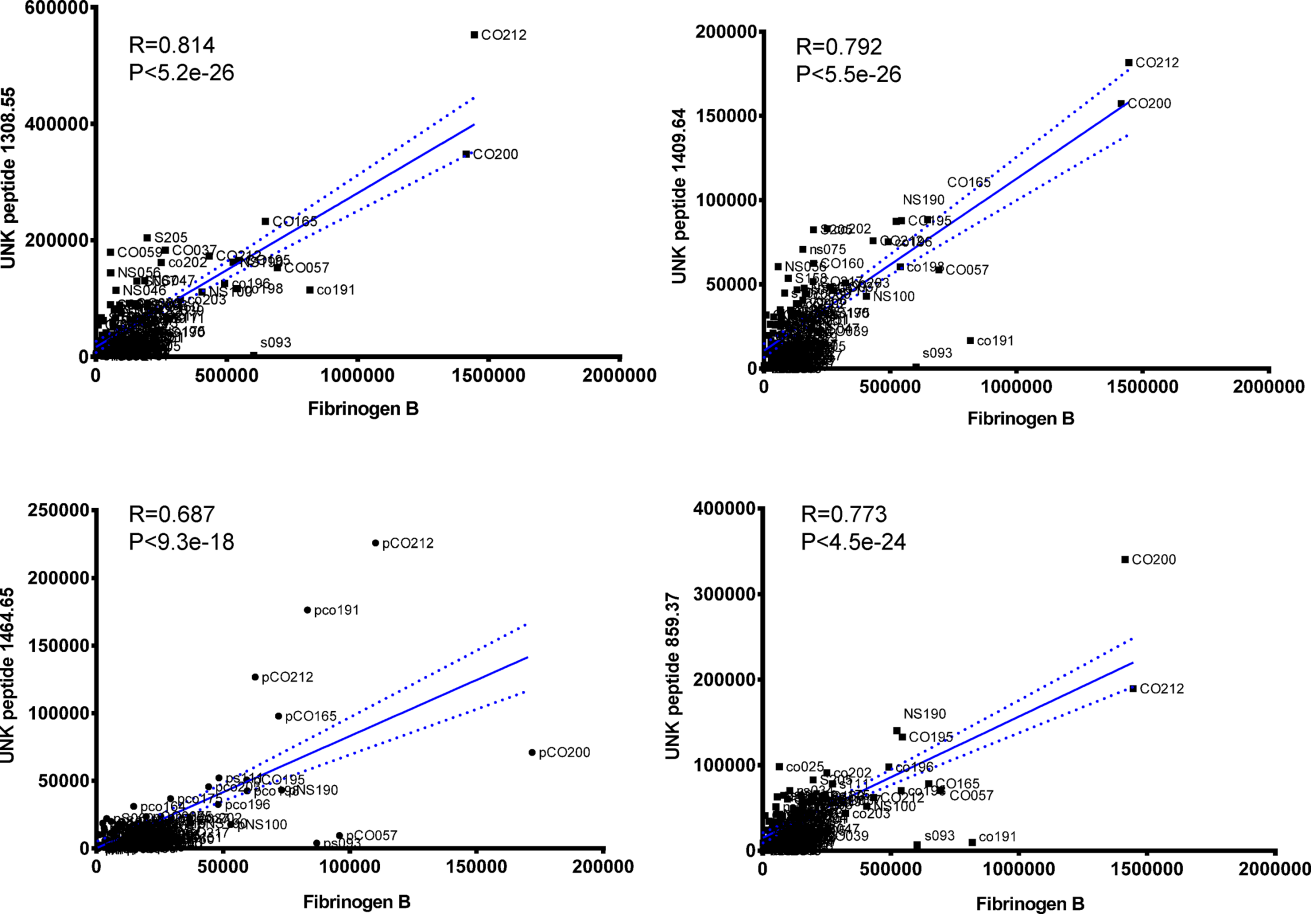
Significant positive correlations of 4 unknown peptides (doubly-charged ions) with des-arginine-fibrinogen peptide B in 115 human serum samples (Pearson correlation coefficient R > 0.67, P < 9.3E-18).

The goal of untargeted metabolite profiling in this study was to discover serum metabolites of known and/or unknown molecular identities that distinguish the population of COPD smokers. Such biomarkers should meet the criteria of discriminating between COPD smokers from healthy smokers, but also exhibit no significant differences between healthy smokers and healthy nonsmokers. **Table 3**summarizes the list of metabolites meeting these criteria. Notably, five peptides were uniquely increased in serum from COPD smokers compared to the other groups. These peptides were all detected by positive ion MS as doubly-charged ions. Fragmentation of the unknown peptides by QTOF revealed that one of these was a modified version of the 14 amino acid fibrinogen peptide B (FPB; EGVNDNEEGFFSAR) (**Fig 2**), wherein the N-terminal glutamate was observed as pyroglutamate and the C-terminal arginine was deleted. Serum products of fibrinogen turnover have been reported be involved in inflammation and wound healing [61], and elevated plasma fibrinogen has previously been associated with reduced pulmonary function and increased risk of COPD [8, 9]. To our knowledge, this is the first report of N-terminal modified and C-terminal truncated fibrinogen peptide B (mFBP) in early-stage COPD smokers. The amino acid sequences of the other four peptides were not solved, but their relative levels were significantly and positively correlated with each other, and with modified fibrinogen peptide B (Pearson coefficient R ≥ 0.68 and P ≤ 9.3 x e^-18^; **Fig 3**).

**Table 3:** Differential metabolites between COPD smokers compared to healthy smokers^1^.

METABOLITEM	Mass	RT	P	FC	P	FC	P	FC
			COPD vs S	COPD vs S	COPD vs NS	COPD vs NS	S vs NS	S vs NS
**Fibrinogen peptide B**	1395.559	8.89	5.56E-03	2.41	7.58E-03	2.38	9.60E-01	0.99
**Peptide 1**	1464.654	10.44	1.14E-02	3.28	1.02E-02	3.74	6.17E-01	1.14
**Peptide 2**	1308.547	9.51	4.75E-03	2.44	8.47E-03	2.23	7.29E-01	0.91
**Peptide 3**	1049.464	9.38	9.04E-04	2.51	1.03E-02	1.89	2.40E-01	0.75
**Peptide 4**	859.3717	8.68	1.12E-02	2.09	6.00E-02	1.65	2.47E-01	0.79
**1,7 Dimethyluric acid**	196.058	2.17	3.72E-02	1.57	9.34E-03	1.8	4.96E-01	1.14
**Myoinositol**	180.062	5.61	3.69E-02	1.33	2.93E-04	1.64	9.87E-02	1.23
**PI(40:7)**	908.542	1.84	4.20E-03	1.24	1.32E-03	1.35	3.92E-01	1.09
**L-Cysteinesulfonic acid**	200.976	1.87	2.33E-02	1.26	2.46E-02	1.23	8.04E-01	0.97
* **Creatinine**	113.056	7.52	3.93E-02	1.15	7.08E-02	1.14	9.03E-01	0.99
**N-Methylnicotinate**	137.049	10.89	5.70E-02	1.72	6.31E-03	2.51	1.72E-01	1.46
* **Unknown 1**	863.402	8.7	6.54E-03	1.94	5.00E-02	1.58	2.62E-01	0.82
**Unknown 2**	627.55	1.85	1.72E-02	1.27	2.83E-04	1.55	6.68E-02	1.22
**Unknown 3**	234.085	9.61	1.53E-03	0.9	6.77E-03	0.87	4.68E-01	0.97
**Unknown 5**	935.513	7.4	1.14E-02	0.81	1.55E-02	0.84	5.68E-01	0.96
**Unknown 6**	592.47	1.61	8.60E-03	0.63	4.28E-02	0.72	4.65E-01	1.14
**Unknown 7**	922.497	1.83	4.73E-02	1.16	1.09E-02	1.27	3.88E-01	1.09
**Unknown 8**	482.105	0.75	1.90E-02	1.38	2.31E-02	1.39	9.48E-01	1.01
**Unknown 9**	834.596	7.19	2.43E-04	1.43	8.84E-02	1.17	7.67E-02	0.82
**Fumarate**	116.011	6.96	1.00E-03	0.72	3.50E-01	0.94	1.00E-02	1.3
**3-hydroxyvalerate**	118.061	1.7	3.40E-03	0.5	4.40E-01	0.89	1.00E-02	1.86
**Unknown 10**	72.056	1.71	4.32E-03	0.59	8.70E-01	0.98	9.47E-03	1.66
**Unknown 11**	496.306	1.05	5.67E-03	0.61	1.27E-01	0.42	4.59E-01	0.70
**Unknown 12**	576.476	1.52	1.07E-02	0.68	2.75E-01	0.88	9.43E-02	1.28
**Unknown 13**	736.464	1.02	1.05E-03	0.43	1.58E-01	0.21	3.82E-01	0.48
**Unknown 14**	290.004	9.28	5.11E-02	1.25	3.10E-01	1.13	4.31E-01	0.90
**Unknown 15**	225.068	0.68	5.05E-02	0.66	2.24E-01	1.25	4.59E-03	1.91
**Unknown 16**	660.262	0.78	1.00E-02	0.68	2.74E-01	0.85	1.57E-01	1.25
* **2-Methylhippuric acid**	193.065	4.36	1.02E-01	1.91	1.54E-02	3.8	1.14E-01	1.99
* **5-Acetylamino-6-amino-3-methyluracil**	198.072	3.57	6.19E-02	1.49	3.97E-03	1.92	2.45E-01	1.29

^1^Underlined metabolites were used for generating prediction model.

*denotes metabolites showing significant correlation with mFBP (Pearson correlation R ≥ 0.34, P ≤ 2.0e-4)

RT = retention; P = p value; FC = fold-change; S = smoker, NS = nonsmokers

In addition to peptides, serum myoinositol, dimethyluric acid and N-methylnicotinate were significantly increased in COPD smokers, as compared with both healthy smokers and non-smokers. Serum cysteinsulfonic acid glycerophosphoinositol, phosphatidylinositol (40:7) and creatinine were also modestly elevated in COPD smokers. Two metabolites, 2-methylhippuric acid and 5-acetylamino-6-amino-3-methyluracil, were markedly increased in COPD smokers compared to healthy non-smokers, and also showed a trend of increase in comparison with healthy smokers, with P values approaching significance. In contrast, several hydrophobic unknowns (with chromatographic retention time consistent with fatty acids and lipids) were mildly decreased in COPD. Notably, the COPD cohort in this study had relatively mild GOLD I and II classified disease, and non-peptide serum metabolite changes were found to be modest compared to healthy smokers, but significant. Except for four metabolites that showed a significant correlation with mFBP (denoted by asterisks in **Table 3**), other non-peptide metabolites were not significantly correlated with mFBP or other observed unsequenced peptides (not shown). Unknown1 (mass 863.40 Da at 8.8 min retention time), creatinine and 2-methylhippurate showed the most significant correlation with mFBP (r = 0.77, P <3.0 x e^-23^; r = 0.47, P<1.3 x e^-7^; and r = 0.47, P<2.5 x e^-7^, respectively). This raises the possibility that COPD-associated changes in non-peptide and peptide metabolites, may have distinct mechanistic implications for COPD pathophysiology.

### Correlation of serum metabolites with clinical parameters

The spirometric parameter FEV1/FVC is the classic hallmark for defining and characterizing the severity of COPD, though it does not always correlate well with health status or disease symptoms. Urine continine and hydroxycotinine reflect the extent of cigarette smoking, but do not correlate with the severity of lung function deficits. In order to find a link between lung dysfunction and altered levels of serum metabolites elicited by smoking, we sought to correlate the relative levels of serum metabolites that distinguish COPD smokers from both healthy smokers and non-smokers with the clinical lung function parameters FEV1/FVC, DLCO, TLC and COHb. **Table 4**lists those metabolites showing a significant Pearson correlation coefficient (P<0.05) with at least one of these lung function parameters. Interestingly, five non-peptide metabolites (N-methylnicotinate, myoinositol, dimethylurate, methylhippurate and 5-acetylamino-6-amino-3-methyluracil) were observed to correlate with all four lung function parameters (P<0.05). In contrast, mFBP and all other non-sequenced peptides selectively and strongly correlate with FEV1/FVC and DLCO (P<0.0003), weakly correlated with COHb, and demonstrated no correlation with TLC. Several other non-peptide metabolites strongly correlated with FEV1/FVC, but exhibited no correlation with COHb and TLC. Serum creatinine and cysteinesulfonic acid showed no significant correlation with any of the lung parameters. Overall, serum metabolites demonstrated a heterogeneous correlation with lung function tests.

**Table 4:** Correlation of serum metabolites with clinical lung parameters (Pearson correlation).

Metabolite	%FEV1/FVC		DLCO		TLC		COHb	
	Pearson R	P value	Pearson R	P value	Pearson R	P value	Pearson R	P value
**N-Methylnicotinate**	-0.3934	1.05E-05	-0.185	0.045	0.3571	7.20E-05	0.3293	0.0003
**Myoinositol**	-0.4385	9.51E-07	-0.2495	0.0071	0.3552	9.81E-05	0.338	0.0002
**Dimethylurate**	-0.3164	0.0006	-0.2609	0.0049	0.2054	0.028	0.2448	0.0087
**2-Methylhippurate**	-0.3524	0.0001	-0.3738	3.87E-05	0.2417	0.0093	0.2684	0.0039
**5-Acetylamino-6-amino-3-methyluracil**	-0.3601	7.72E-05	-0.3279	0.0003	0.2534	0.0063	0.3144	0.0007
**863.40@8.7**	-0.3636	7.00E-05	-0.2891	0.0018	0.0004137	NS	0.1252	NS
**627.55@1.85**	-0.2415	0.0084	-0.2297	0.012	-0.004	NS	0.211	0.02
**592.47@1.67**	0.2821	0.0023	-0.1535	NS	0.009827	NS	0.02193	NS
**284.061@8.88**	0.239	0.0091	0.2421	0.0083	-0.05	NS	-0.11	NS
**234.085@9.61**	0.2473	0.0069	0.1205	NS	-0.05	NS	-0.06	NS
**Fibrinogen peptide B**	-0.3016	0.0009	-0.3659	4.61E-05	-0.00968	NS	0.2393	0.0094
**Peptide 1464.65@10.44**	-0.326	0.0003	-0.5185	1.81E-09	0.1385	NS	0.2422	0.0085
**Peptide 859.371@8.68**	-0.3763	4.00E-05	-0.3096	0.0003	0.003102	NS	0.1267	NS
**Peptide 1308.547@9.51**	-0.3794	2.89E-05	-0.433	1.34E-06	0.07092	NS	0.1768	0.0558
**Peptide 1049.464@9.38**	-0.4115	7.23E-06	-0.3942	1.86E-05	0.04195	NS	0.1725	0.075

### Serum metabolite prediction model for COPD

**Fig 4:**
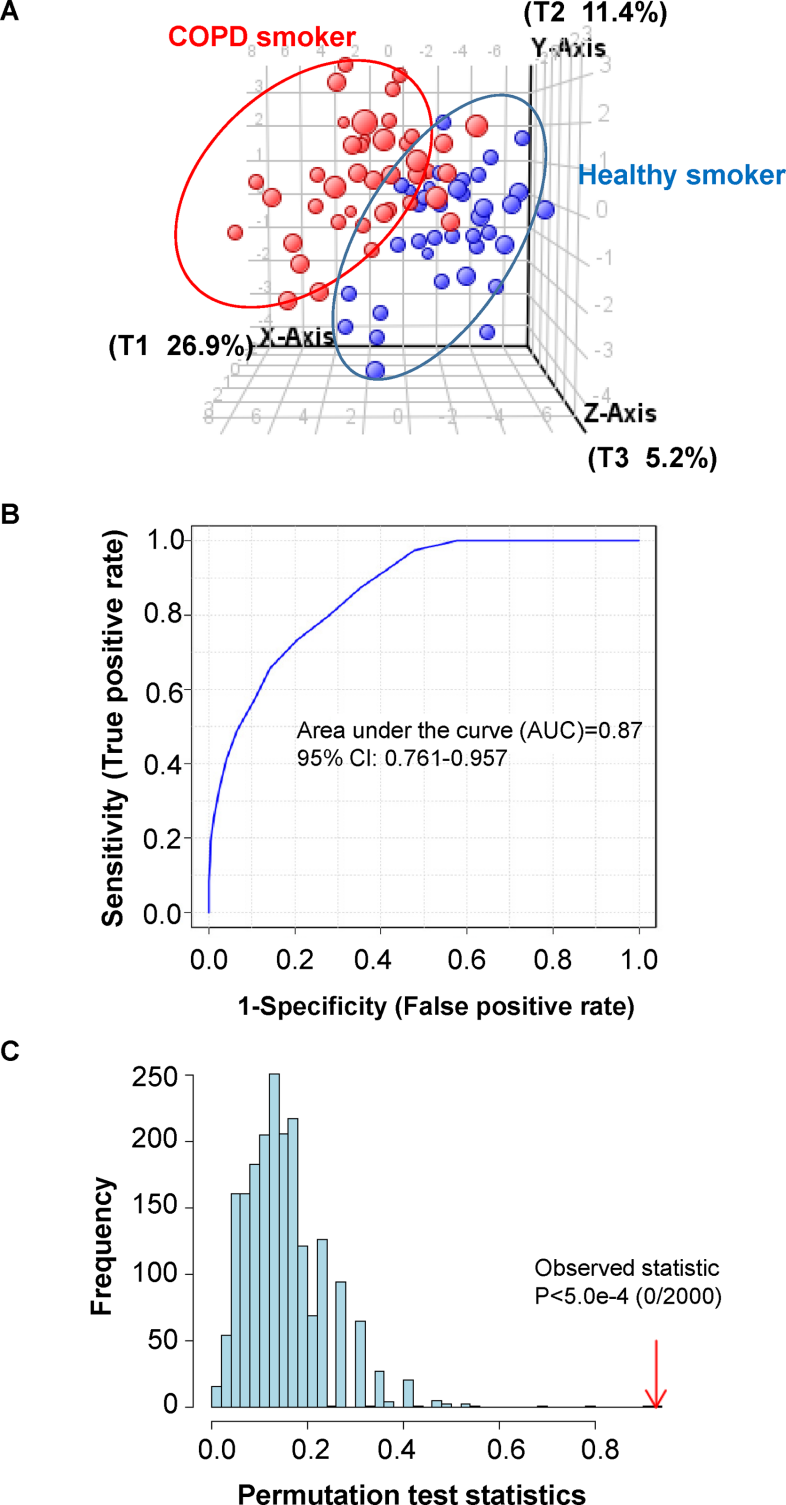
Characterization of a PLS-DA prediction model for identification COPD smokers, based on 23 serum metabolites. (A) Three-dimensional score plot of PLS-DA classification model showing the partial separation of COPD smokers from healthy smokers. (B) Diagnostic statistics for the presented PLS-DA model, as area under the receiver operator characteristics curve (AUC). The AUC was 0.87 with a 95% confidence interval of 0.761–0.957 for the constructed model. (C) The location of original between-sum squares and the within-sum squares (B/W-ratio), relative to the distribution histogram of 2000 permuted B/W ratios. The B/W ratio of the original classification assignment (COPD smokers vs. healthy smoker.) was further to the right of the distribution, with a permutation P value less than 5.0 x e^-4^, affirming the statistical significance and validity of the PLS-DA model.

A serum metabolite-based prediction model for early early-stage COPD may suggest pathophysiological mechanisms and have predictive power beyond that offered by lung function parameters alone. Accordingly, we sought to identify a predictive serum metabolite signature for GOLD stage I/II COPD. Because non-smokers with no prior exposure to cigarette smoking and no detectable nicotine metabolites are unlikely to develop COPD, non-smokers were excluded from COPD model generation. Considering 23 metabolites that distinguished COPD smokers from healthy smokers (underlined metabolites in **Table 3**), we conducted a partial least square discriminant analysis (PLS-DA) to classify the COPD smokers and healthy smokers, employing Agilent MassProfiler Professional 13.0 software. The PLS-DA score plot shows a marked separation of COPD smokers from healthy smokers (**Fig 4A**). The sensitivity of the constructed model was calculated from the ratio of predicted true COPD smokers vs. total COPD smokers, whereas specificity of the model was calculated as the ratio of predicted true healthy smokers to the total number of healthy smokers. Applying this quantitative approach, the classification sensitivity was found to be 36 out of 41 COPD smokers (87.8%), and the classification specificity was 32 out of 37 healthy smokers (86.5%). Based on these findings, the overall accuracy of classification was 87.2%. The receiver operator characteristics (ROC) were obtained by plotting the sensitivity against specificity at different discriminant thresholds. The area under the ROC, commonly used as a diagnostic statistic for PLS-DA models, was 0.87 (**Fig 4B**). ROC curves were generated using *ROC Curve Explorer and Tester* (ROCCET, http://www.roccet.ca/ROCCET/), using the same set of data for which *Monte Carlo Cross Validation* (MCCV) was applied via balanced subsampling. In each MCCV analysis, two thirds of the samples were used to evaluate the feature importance. The 23 differentially expressed COPD serum metabolites were then used to build classification models that were validated based on 1/3 of the samples left out. The procedure was repeated multiple times to calculate the performance and confidence interval for the model.

PLS-DA is prone to data over-fitting, and therefore the generated models need to be validated by assessing whether the observed separation is indeed statistically significant, or due to random noise. To eliminate the random chance that the constructed model has discriminating power for COPD smokers vs. healthy smokers, a permutation test was performed using non-proprietary MetaboAnalyst 3.0 software, involving 2,000 permutated class labels–this tested the assumption that there is no difference between COPD smokers and healthy smoker groups. In each permutation, a PLS-DA model was built between the data (X) and the permuted class labels (Y), using the optimal number of components determined by cross-validation of the model based on the original class assignment. The ratio of between-sum squares and the within-sum squares (B/W-ratio) for the prediction of each model was calculated. **Fig 4C** shows the location of original B/W ratio, relative to the distribution histogram of 2,000 permuted B/W ratios. The B/W ratio of the original classification assignment (COPD smokers vs. healthy smokers) was further away from the right of the distribution with a permutation P value of less than 5.0 x e^-4^, indicating that the PLS-DA model of COPD- and healthy- smoker classification is statistically significant.

### Validation of the COPD classification model

We sought to test the efficacy of the 23 metabolite signature for prediction of early-stage COPD in an independent group of subjects. As shown in S1 Table, applying the 23 serum metabolite model to an independent group of 10 early-stage COPD patients correctly classified these subjects as having COPD in 80% of cases. Further, in a cohort of 19 COPD patients who quit smoking for ≥ 3 months, this classification algorithm recognized the COPD metabotype in 14/19 subjects in accord with their active COPD.

## Discussion

In attempt to uncover biomarkers that identify early-stage (GOLD I/II) COPD, we performed untargeted LC/MS profiling in a cohort of 118 healthy smokers, COPD smokers and non-smokers with well-characterized clinical lung function parameters. We report here the identity of circulating serum metabolites as potential COPD biomarkers. To our knowledge, this is the first comprehensive untargeted metabolite profiling for serum markers of COPD, comparing non-smokers, healthy smokers and early-stage COPD smokers. This analysis identified a group of 23 non-peptide and peptide metabolites, some of which were not directly correlated with any lung function parameters, potentially providing pathophysiological information over and above that obtained from clinical testing. Efficacy of this putative signature for early-stage COPD was provided by an independent validation cohort in which COPD was predicted with 80% success.

One biomarker identified in serum distinguishing COPD smokers from smokers was a modified form of the fibrinogen-derived peptide, FPB. Fibrinogen is an acute phase plasma protein that has emerged as a promising biomarker in COPD [8, 9, 62, 63]. Elevated levels of plasma fibrinogen have been associated with pulmonary dysfunction, coronary heart disease [63] and hospitalization of COPD patients [9]. In blood, thrombin converts fibrinogen to fibrin in two sequential steps [64], ultimately resulting in equimolar quantities of FPB and fibrin II. Fibrin II is a mediator of thrombosis, polymerizing together with platelets it forms a hemostatic plug or clot over wound sites [65]. The 14-amino acid FPB is susceptible to cleavage by carboxypeptidase in blood, where removal of the C-terminal arginine yields des-arginine FPB. Therefore, measurement of des-arginine FPB indirectly signifies *in vivo* fibrin II production. Fibrin clot propensity was previously found to be altered in patients with COPD [66]. In accord with this prior finding [66]; our untargeted metabolite profiling revealed that N-terminally modified des-arginine FPB metabolite (pyroEGVNDNEEGFFSA) was elevated in the circulating blood of early-stage COPD subjects, as compared to healthy smokers with a similar smoking history and extent of cigarette exposure. Further, we demonstrated a significant positive correlation between the level of mFBP and the lung function parameters FEV1/FVC% and DLCO in all study subjects. This elevation in mFBP levels suggests that coagulation and thrombogenesis may play a role in COPD smokers. Indeed, quantitative exposure to passive smoke has been shown to positively correlate with blood coagulation activity [67]. The association between acute inflammation, endothelial activation and clotting initiation has been demonstrated in COPD exacerbations [68]. In addition to mFBP, three other unknown peptides were increased in COPD smokers only. Based on the significant correlation with each other and with mFBP, we postulate that these peptides may also belong to the fibrinogen family and /or the thrombotic cascade.

Despite a significantly increased level of circulating mFBP and other peptides in COPD patient serum, the presence of peptides alone did not possess predictive power to accurately identify early stage COPD smokers (AUC ≈ 0.75, not shown). Interestingly, the 4 observed peptide biomarkers shared a negative correlation with the lung function parameters FEV1/FVC% and DLCO, but not with the lung parameters TLC and COHb. In contrast, non-peptide small molecule serum metabolites, including myoinositol, dimethylurate, N-methylnicotinate, and 5-acetylamino-6-amino-3-methyluracil were all significantly correlated with each of the 4 lung function parameters obtained in our patient cohort. Among the non-peptide metabolites, myoinositol evidenced the most significant correlation with clinical parameters. Myoinositol is a cyclic sugar alcohol that functions in glycerophospholipid metabolism and inositol phosphate metabolism, also serving as an osmolyte. Moreover, inositol phosphoglycans comprise a major family of intracellular signaling molecules, including the mediation of insulin action [69]. Furthermore, myoinositol has been used as chemoprevention drug in clinical trials, acting to inhibit Akt and extracellular signal-regulated kinase (ERK) in bronchial lesions from heavy smokers [70]. *In vitro* studies have shown that myoinositol decreases endogenous and tobacco carcinogen-induced activation of Akt and ERK in immortalized human bronchial epithelial cells [70]. In COPD patient’s sera, we observed that both myoinositol and the inositol-derived plasma membrane phospholipid, glycerophosphoinositol [PI (40:7)] was increased compared to healthy smokers and healthy non-smokers, and elevated levels of glycerophosphoinositol lipids were found in healthy smokers compared to healthy non-smokers. These results suggest an altered pattern of phosphatidylinositol metabolism in smokers. Consistent with this observation, smoking-derived toxins have been related to the activation of PI3K signaling pathway [71–73], altering cell growth, proliferation, survival, and motility in COPD smokers. Thus, increased myoinositol in COPD smokers may be the consequence of feedback regulation of activated PI3K. Notably, high serum myoinositol in COPD can suppress the synthesis of phosphatidylglycerol an important surfactant in the alveolar wall which has been show to decrease in COPD and patients with cystic fibrosis [74].

Elevated levels of serum metabolite N-methylnicotinate, 1,7dimethylurate and 5-acetylamino-6-amino-3-methyluracil (AAMU) in COPD subjects were unexpected, but intriguing. These three metabolites are all related to caffeine metabolism. N-methylnicotinate (aka trigonelline) is a pyridine alkaloid and metabolite of caffeine, as well as a phytoestrogen, in coffee beans [75, 76]. 1,7 dimetylureate is a metabolic product of caffeine, whose synthesis is primarily catalyzed by CYP2A6. AAMU is also a major caffeine metabolite, synthesized by N-acetyltransferase 2. Notably, these urinary caffeine metabolites have been used to assess the enzymatic activities of xanthine oxidase (XO) [77–79] and N-acetyltransferase 2 (NAT2) and cigarette smoking has been shown to induce cytochrome P450-mediated alkaloid metabolism [80]. Taken together, these findings suggest an acceleration of caffeine metabolism in COPD smokers, especially for the downstream caffeine metabolites resulting from activated CYP2A6. This is the first untargeted serum profiling finding that points to a potential upregulation of caffeine metabolism in COPD smokers.

In summary, our untargeted serum metabolite profiling revealed metabolite biomarkers of early-stage COPD that are indicative of thrombosis, altered cell signaling and metabolic changes. Based on 23 serum metabolites, the prediction of COPD amongst a mixed group of both healthy and COPD smokers had an overall 87% accuracy and was found to be 80% correct when predicting COPD in an independent population of COPD smokers. Combined with clinical parameters, the identified serum markers offer the potential to improve sensitivity and specificity for predicting and monitoring COPD prognosis in cigarette smokers.

## Supporting Information

S1 Table Prediction of COPD in subjects with COPD who are either active smokers (COPDact) and who have quit smoking for ≥ 3 months (COPDquit). (DOCX)

## Abbreviations

COPD: Chronic obstructive pulmonary disease
LC/MS: liquid chromatography mass spectrometry
mFPB: pyroglutamate des-arginine fibrinopeptide B
GOLD: Global Initiative for Chronic Obstructive Lung Disease
FEV1: forced expiratory volume in 1 second
FVC: forced vital capacity
TLC: total lung function
DLCO: diffusing capacity of the lung for carbon monoxide
COHb: carboxyhemoglobin
