# Supplementary material for: Serum Metabolite Biomarkers Discriminate Healthy Smokers from COPD Smokers
Source: PLoS One. 2015 Dec 16;10(12):e0143937. doi: 10.1371/journal.pone.0143937 (PMC4682670; doi:10.1371/journal.pone.0143937)
Supplement: S1 Table — (DOCX) [file pone.0143937.s001.docx]

**Supporting Information.** Original MS ion abundance data are provided in Excel spreadsheet format, used for calculation of differentially-expressed metabolites in study subjects (i.e., non-smokers, healthy-smokers, COPD-smokers and Quit-smokers).

**Table S1. Prediction of COPD in subjects with COPD who are either active smokers (COPDact) and who have quit smoking for ≥ 3 months (COPDquit).**

| Sample Name | Predicted |
| --- | --- |
| COPDact-1 | [Non-COPD] |
| COPDact-10 | [COPD] |
| COPDact-2 | [COPD] |
| COPDact-3 | [Non-COPD] |
| COPDact-4 | [COPD] |
| COPDact-5 | [COPD] |
| COPDact-6 | [COPD] |
| COPDact-7 | [COPD] |
| COPDact-8 | [COPD] |
| COPDact-9 | [COPD] |
| COPDquit-1 | [Non-COPD] |
| COPDquit-10 | [Non-COPD] |
| COPDquit-11 | [COPD] |
| COPDquit-12 | [COPD] |
| COPDquit-13 | [COPD] |
| COPDquit-14 | [COPD] |
| COPDquit-15 | [COPD] |
| COPDquit-16 | [COPD] |
| COPDquit-17 | [COPD] |
| COPDquit-18 | [COPD] |
| COPDquit-2 | [Non-COPD] |
| COPDquit-3 | [Non-COPD] |
| COPDquit-4 | [Non-COPD] |
| COPDquit-5 | [COPD] |
| COPDquit-6 | [COPD] |
| COPDquit-7 | [COPD] |
| COPDquit-8 | [COPD] |
| COPDquit-9 | [COPD] |
